# Expression profile and prognostic role of sex hormone receptors in gastric cancer

**DOI:** 10.1186/1471-2407-12-566

**Published:** 2012-12-02

**Authors:** Lu Gan, Jian He, Xia Zhang, Yong-Jie Zhang, Guan-Zhen Yu, Ying Chen, Jun Pan, Jie-Jun Wang, Xi Wang

**Affiliations:** 1 Department of Oncology, Changzheng Hospital, Second Military Medical University, Shanghai, China; 2 Department of Pathology, Changhai Hospital, Second Military Medical University, Shanghai, China

**Keywords:** Gastric cancer, Estrogen receptor alpha, Estrogen receptor beta, Progesterone receptor, Androgen receptor, Prognosis

## Abstract

**Background:**

Increasing interest has been devoted to the expression and possible role of sex hormone receptors in gastric cancer, but most of these findings are controversial. In the present study, the expression profile of sex hormone receptors in gastric cancer and their clinicopathological and prognostic value were determined in a large Chinese cohort.

**Methods:**

The mRNA and protein expression of estrogen receptor alpha (ERα), estrogen receptor beta (ERβ), progesterone receptor (PR), and androgen receptor (AR) in primary gastric tumors and corresponding adjacent normal tissues from 60 and 866 Chinese gastric cancer patients was detected by real-time quantitative PCR and immunohistochemistry method, respectively. The expression profile of the four receptors was compared and their associations with clinicopathological characteristics were assessed by using Chi-square test. The prognostic value of the four receptors in gastric cancer was evaluated by using univariate and multivariate Cox regression analysis.

**Results:**

The presence of ERα, ERβ, PR, and AR in both gastric tumors and normal tissues was confirmed but their expression levels were extremely low except for the predominance of ERβ. The four receptors were expressed independently and showed a decreased expression pattern in gastric tumors compared to adjacent normal tissues. The positive expression of the four receptors all correlated with high tumor grade and intestinal type, and ERα and AR were also associated with early TNM stage and thereby a favorable outcome. However, ERα and AR were not independent prognostic factors for gastric cancer when multivariate survival analysis was performed.

**Conclusions:**

Our findings indicate that the sex hormone receptors may be partly involved in gastric carcinogenesis but their clinicopathological and prognostic significance in gastric cancer appears to be limited.

## Background

Hormonal therapy is one of the major modalities of systemic treatment for hormone-dependent tumors such as breast cancer and prostate cancer [1,2]. The principle of hormonal therapy is that the sex hormones, estrogen or androgen, stimulate specific hormone-dependent cancer cells to grow and spread. Deprivation on the synthesis of such hormones or blocking the hormone signaling pathways can induce tumor recession. Particularly, it is well demonstrated that the hormone receptors are pivotal targets for treatment of hormone-dependent tumors. Tamoxifen, an estrogen antagonist competitively binding to estrogen receptor (ER), has obtained great success in the treatment of ER-positive breast cancer in the past several decades [1-6]. The success of tamoxifen has prompted investigators to evaluate the possible role of hormone receptors in a variety of other tumors derived from “nontarget” organs and determine the possibility of hormonal therapy for these tumors, including gastric cancer [7,8].

Since 1983, a few of studies have examined the expression of ER in gastric cancer. However, considerable controversy is raised as to the expression level of ER and its prognostic value in gastric cancer [9-27]. More detailedly, a few studies show that ER alpha (ERα) is absent in gastric cancer but ER beta (ERβ) is expressed in abundance, whereas others indicate that the two ER isoforms are both expressed [22-27]. Furthermore, some authors find ER expression is correlated with poor differentiation, advanced stage, and adverse outcome while others suggest the opposite [17,19,23,24,26,27]. Additionally, the roles of progesterone receptor (PR) and androgen receptor (AR) in gastric cancer are poorly defined [28]. In the present study, the expression profile of four sex hormone receptors, ERα, ERβ, PR, and AR, was determined in gastric tumors and corresponding normal tissues from a large Chinese cohort, and their clinicopathological and prognostic value was assessed.

## Methods

### Patients and tissue samples

A total of 1072 patients underwent gastrectomy for histopathologically confirmed gastric carcinoma in Changhai Hospital, Second Military Medical University, Shanghai, China, from 2000 through 2005. Patients without sufficient tissue samples or necessary clinicopathological information, or patients suffered from double primary tumors or remnant gastric cancer, or those died within two months of surgery, were all excluded and thus 866 eligible patients were enrolled. The patients were followed up every 6 months until death or study end (March 30 2010), except for those lost to follow-up. The tumor tissues and their adjacent normal tissues from these patients were routinely fixed in 10% buffered formalin and blocked in paraffin, ready to tissue microarray construction. In addition, 60 pairs of fresh gastric tumors and their matched normal mucosa were obtained. The fresh tissue samples were prepared carefully within 15 min of excision, stabilized in RNA*later*® solution (Ambion, Austin, TX, USA) at 4°C overnight and preserved at −20°C until RNA extraction. All patients enrolled were naïve for any anticancer therapy. All tissue specimens were obtained with patient informed consent, and the protocol was approved by Institutional Review Board of Second Military Medical University.

### Total RNA preparation and reverse transcription

Total RNA was extracted from the RNA*later*®-stabilized tissue samples using an RNAqueous®-4PCR kit (Cat# AM1914, Ambion, Austin, TX, USA) according to the manufacturer’s protocol. Complementary DNA (cDNA) was synthesized from total RNA with use of a High Capacity cDNA Reverse Transcription Kit (PN4374966, Applied Biosystems, Foster City, CA), according to the manufacturer’s instructions. The reaction was incubated in an ABI 2720 Thermocycler (Applied Biosystems) for 10 min at 25°C, 120 min at 37°C, and 5 min at 85°C. cDNA samples were stored at −20°C before real-time PCR amplification.

### Real-time quantitative PCR

Real-time quantitative PCR was performed with an ABI PRISM® 7900HT Sequence Detection System (Applied Biosystems) using the Power SYBR® Green PCR Master Mix kit (PN4367659, Applied Biosystems) as described by the manufacturer. A total reaction volume of 50 μl contained 5 μl of cDNA template corresponding to 100 ng of total RNA, 25 μl of 2 × Power SYBR® Green PCR Master Mix, 1 μl forward primer of 10 μM, 1 μl reverse primer of 10 μM and 18 μl ddH_2_O. Negative controls included water instead of cDNA in the PCR reaction and addition of RNA instead of cDNA, and β-actin was used as an endogenous control. The primer sequences were as follows: ERα 5^′^-TCCTGATGATTGGTCTCGTCT-3^′^ (forward) and 5^′^-ACATTTTCCCT GGTTCCTGTC-3^′^ (reverse), ERβ 5^′^-AGTCTGGTCGTGTGAAGGATG-3^′^ (forward) and 5^′^-ACTTCTCTGTCTCCGCACAAG-3^′^ (reverse), PR 5^′^-ACACCTCCAGTTCTTTGCTGAC-3^′^ (forward) and 5^′^-ATTCTTTCATCCGCTGTTCATT-3^′^ (reverse), AR 5^′^-ATTGTCCATCTTGTCGTCTTCG-3^′^ (forward) and 5^′^-AGCCTCTCCTTCCTCCTGTAGT-3^′^ (reverse), and β-actin 5^′^-TGTTACAGGAAGTCCCTTGC-3^′^ (forward) and 5^′^-AAGCAATGCTATCACCTCCC-3^′^ (reverse). All primers were synthesized by Sangon Biotech Co. Ltd., Shanghai, China. The amplification was run at 95°C for 10 min followed by 40 cycles of 95°C for 15 sec and 60°C for 1 min. All samples were run in triplicate, and data were analyzed by use of the Sequence Detection System (SDS) Software Version 2.3 (Applied Biosystems). The specificity of amplification reaction was confirmed by analyzing the corresponding dissociation curves. The quantification of sex hormone receptors was normalized to β-actin expression using the 2^-ΔΔCt^ method.

### Tissue microarray construction and immunohistochemistry

Tissue microarrays were constructed from formalin-fixed and paraffin-embedded archival tissue blocks using a tissue arrayer (Beecher Instruments, Silver Spring, MD) according to the previous description [29,30]. For each of 866 patients, duplicate gastric tumor cylinders and at least one matched adjacent normal mucosa cylinder with a diameter of 1.5 mm were arrayed and consecutive 4 μm sections were cut. Immunohistochemistry assay for ERα, ERβ, PR, and AR was performed using an UltraSensitive™ SP kit (#9710, Maixin, Fuzhou, China) according to the manufacturer’s instructions. Briefly, the tissue microarray sections were deparaffinized in xylene, rehydrated with graded ethanol, and subjected to antigen retrieval in citrate buffer (pH 6.0) in a high-pressure cooker. The sections were subsequently blocked for endogenous peroxide activity with 3% hydrogen peroxide, treated with preimmune goat serum to block nonspecific binding sites, and then incubated with the primary mouse monoclonal antibodies against ERα (clone 33, ab2746, Abcam; 1:50), ERβ (clone 14C8, ab288, Abcam; 1:100), PR (clone PR-AT 4.14, ab2764, Abcam; 1:100), and AR (clone AR 441, ab9474, Abcam; 1:200), respectively. After an overnight incubation at 4°C, the sections were washed and incubated with a secondary biotinylated anti-mouse/rabbit antibody. The immunostaining was visualized with a diaminobenzidine detection kit (DAB-0031, Maixin) and then the sections were counterstained with hematoxylin, dehydrated, cleared, and coverslipped. Human breast cancer tissue overexpressing ERα, ERβ, and PR, and prostate cancer tissue overexpressing AR were used as positive controls. Sections incubated without primary antibody were also included in each staining experiment as negative controls.

### Evaluation of immunostaining

Brown cytoplasmic and/or nuclear staining in the gastric cancer cells or adjacent normal epitheliums was considered to be positive. The signal was quantified by the Allred score system which represented the estimated intensity and proportion of positive-staining cells [31]. A score ≥3 was designated as positive expression and a score of 0 or 2 was regarded as negative. The immunostaining sections were viewed by two pathologists independently using an Olympus CX31 microscope (Olympus, Japan).

### Statistical analysis

The expression difference of sex hormone receptors between gastric tumors and corresponding normal tissues was determined by the Wilcoxon matched-pairs signed-rank test or Chi-square test where appropriate. Correlations were computed using the Spearman rank test. The associations between expression of sex hormone receptors and clinicopathological characteristics were tested using Chi-square test. The probability of survival was estimated by Kaplan-Meier method and compared by log-rank test. The prognostic role of sex hormone receptors in gastric cancer was identified using univariate and multivariate Cox model. All *P* values were two-sided and less than 0.05 was considered statistically significant. Statistical analyses were performed by the SPSS 15.0 for windows (SPSS, Chicago, IL, USA).

## Results

### Expression profile of sex hormone receptors in gastric cancer

**Figure 1:**
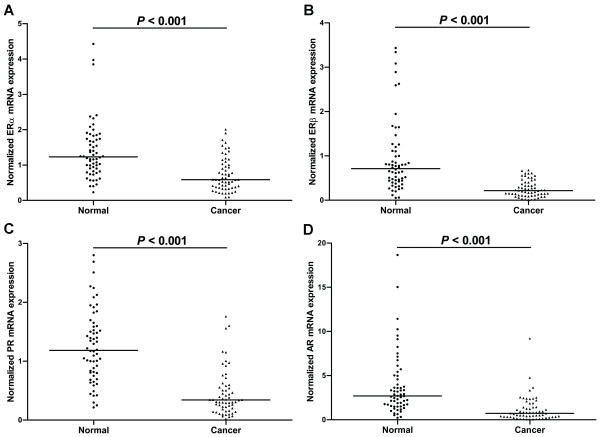
**Scatter plots of (A) ERα, (B) ERβ, (C) PR, and (D) AR mRNA levels in gastric tumors and corresponding adjacent normal mucosa (*n* = 60).** The line indicates the median value. The mRNA levels were normalized to β-actin and statistical differences were determined using Wilcoxon matched-pairs signed-rank test.

Real-time quantitative PCR showed that the mRNAs of ERα, ERβ, PR and AR were all detected in all 60 pairs of gastric tumors and their matched normal mucosa. Furthermore, the mRNA levels of the four receptors in gastric tumors were all significantly decreased compared to those in their matched normal mucosa (Figure 1).

**Figure 2:**
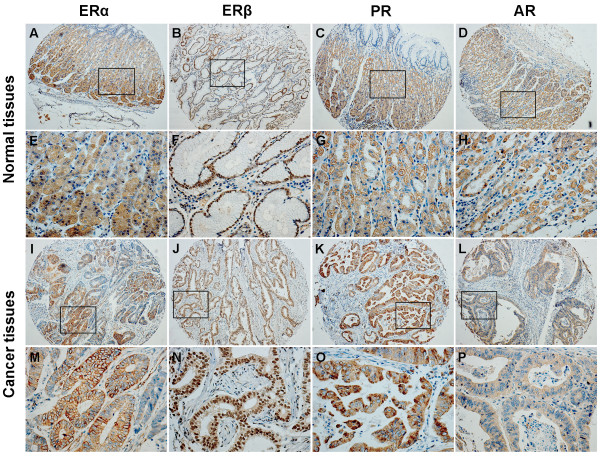
**Representative immunostaining of sex hormone receptors in gastric tumors and corresponding adjacent normal tissues.** Positive staining of (**A**, **E**) ERα, (**B, F**) ERβ, (**C, G**) PR, and (**D**, **H**) AR in normal tissues, and positive staining of (**I**, **M**) ERα, (**J, N**) ERβ, (**K, O**) PR, and (**L**, **P**) AR in gastric tumors is shown. Original magnification, ×100 for (**A**) through (**D**) and (**I**) through (**L**); ×400 for (**E**) through (**H**) and (**M**) through (**P**).

Immunohistochemistry assay based on 866 Chinese patients further demonstrated the expression of ERα, ERβ, PR and AR proteins. Unlike the typical nuclear expression in breast and prostate cancer tissues as positive controls (see Additional file 1: Figure S1), the four sex hormone receptors all presented a cytoplasmic/nuclear staining pattern (Figure 2). However, ERα, PR and AR immunostaining was mainly localized in the cytoplasm while ERβ immunostaining was ubiquitously observed in the nucleus for both normal epithelium and gastric cancer cells (Figure 2). After exclusion of inevaluable cases due to tissue loss or inadequate tissue, the positive rates of ERα, ERβ, PR and AR expression in normal tissues were 38.3%, 97.3%, 30.5%, and 52.7%, and the positive rates of the four receptors in gastric tumor were 12.0%, 91.9%, 23.3%, and 33.0%, respectively. The protein level of each receptor in gastric tumor was all significantly lower than that expressed in normal gastric mucosa (*P* < 0.001).

### Correlations among expression of sex hormone receptors in gastric cancer

Table 1 shows the Spearman correlations among expression of the four sex hormone receptors on mRNA and protein levels. Strong correlations of mRNA expression between ERα and ERβ, ERα and PR, and ERβ and PR were revealed, while no significant correlations between AR and the other three receptors were detected. On protein level, significant correlations were observed in all pairwise comparison among immunostaining scores of the four receptors. However, these correlation coefficients were so small (*r* < 0.4) that only extremely weak correlations among the four receptors were found.

**Table 1:** Correlations among expression of sex hormone receptors in gastric cancer

**Correlation**	**mRNA expression**		**Protein expression**	
	***r***	***P*†**	***r***	***P*†**
ERα *vs* ERβ	0.795	<0.001	0.136	<0.001
ERα *vs* PR	0.756	<0.001	0.083	0.016
ERα *vs* AR	0.328	0.437	0.171	<0.001
ERβ *vs* PR	0.714	<0.001	0.132	<0.001
ERβ *vs* AR	0.492	0.231	0.329	<0.001
PR *vs* AR	0.186	0.734	0.098	0.005

*ERα*, estrogen receptor alpha; *ERβ*, estrogen receptor beta; *PR*, progesterone receptor; *AR*, androgen receptor; *r*, Spearman rank correlation coefficients. †Spearman rank correlation test.

### ERα and AR expression correlates with tumor grade, Lauren type, and TNM stage of gastric cancer

Possible associations of ERα, ERβ, PR and AR expression with available clinicopathological characteristics of 866 gastric cancer patients are presented in Table 2. The protein expression of ERα and AR was closely associated with tumor grade, Lauren type, T classification, and N classification (*P* < 0.001), respectively, and consequently correlated with TNM stage (*P* < 0.001). Importantly, positive staining of ERα and AR was more frequently observed in patients with better differentiated tumors, intestinal type, and earlier TNM stage. Either for ERβ or for PR, only correlations between the positivity and the tumor grade and Lauren type were noticed (*P* < 0.05). No significant associations were found between expression of ERβ and PR and other clinicopathological characteristics.

**Table 2:** Association between expression of sex hormone receptors and clinicopathological characteristics in gastric cancer

**Variable**	**Total patients (*n*** **= 866)**	**Evaluable patients†**							
		**ERα (*n*** **= 848)**		**ERβ (*n*** **= 823)**		**PR (*n*** **= 851)**		**AR (*n*** **= 843)**	
		**Positive No. (%)**	***P*‡**	**Positive No. (%)**	***P*‡**	**Positive No. (%)**	***P*‡**	**Positive No. (%)**	***P*‡**
Sex			0.496		0.166		0.522		0.313
Female	261	29 (10.9)		221 (89.8)		58 (21.9)		81 (30.6)	
Male	605	73 (12.5)		535 (92.7)		140 (23.9)		197 (34.1)	
Age, years			0.837		0.187		0.523		0.254
≤40	70	6 (8.8)		57 (87.7)		12 (17.6)		16 (24.2)	
≤50	148	19 (13.2)		126 (93.3)		30 (20.7)		46 (32.2)	
≤65	328	39 (12.0)		283 (90.1)		79 (24.5)		102 (31.9)	
>65	320	38 (12.2)		290 (93.9)		77 (24.4)		114 (36.3)	
Tumor site§			0.839		0.700		0.433		0.605
Upper	138	19 (13.8)		123 (91.8)		25 (18.2)		52 (38.0)	
Middle	263	30 (11.8)		224 (90.3)		63 (24.3)		81 (31.9)	
Lower	416	46 (11.3)		368 (92.9)		100 (24.6)		129 (32.0)	
Diffuse	49	7 (14.3)		41 (91.1)		10 (20.4)		16 (32.7)	
Tumor size, cm			0.166		0.316		0.425		0.127
≤2	133	22 (17.1)		117 (95.9)		28 (22.0)		52 (42.3)	
≤3	160	21 (13.7)		134 (89.9)		29 (18.6)		50 (32.3)	
≤5	273	30 (11.1)		243 (91.7)		67 (24.7)		84 (31.3)	
>5	300	29 (9.8)		262 (91.3)		74 (24.9)		92 (31.0)	
Tumor grade			<0.001		0.030		0.001		<0.001
Well	48	15 (31.9)		53 (96.4)		19 (40.4)		22 (48.9)	
Moderate	286	47 (16.5)		274 (94.5)		77 (27.1)		127 (44.9)	
Poor	532	40 (7.8)		429 (89.7)		102 (19.6)		129 (25.0)	
Lauren type			0.012		0.010		0.017		<0.001
Intestinal	535	76 (14.4)		481 (93.0)		139 (26.3)		197 (37.6)	
Diffuse	297	21 (7.4)		250 (91.2)		55 (19.0)		68 (23.9)	
Mixed	34	5 (14.7)		25 (78.1)		4 (11.8)		13 (38.2)	
T classification¶			<0.001		0.195		0.818		<0.001
pT1	132	33 (26.0)		114 (95.0)		28 (21.7)		61 (48.8)	
pT2	142	19 (13.9)		125 (94.7)		36 (26.3)		49 (36.6)	
pT3	528	44 (8.4)		463 (90.8)		120 (23.0)		147 (28.2)	
pT4	64	6 (9.7)		54 (88.5)		14 (22.2)		21 (33.9)	
N classification¶			<0.001		0.574		0.340		0.001
pN0	309	67 (22.3)		272 (93.2)		76 (25.0)		125 (41.9)	
pN1	314	20 (6.5)		275 (90.2)		63 (20.7)		85 (28.0)	
pN2	191	13 (6.9)		165 (92.7)		43 (22.6)		52 (27.4)	
pN3	52	2 (3.9)		44 (91.7)		16 (30.8)		16 (31.4)	
TNM stage¶			<0.001		0.173		0.535		<0.001
I	187	44 (24.4)		165 (95.4)		48 (26.2)		85 (47.8)	
II	158	21 (13.5)		138 (90.8)		39 (25.3)		53 (35.1)	
III	294	23 (8.0)		253 (89.7)		61 (21.0)		74 (25.3)	
IV	227	14 (6.3)		200 (92.6)		50 (22.3)		66 (29.7)	

*ERα*, estrogen receptor alpha; *ERβ*, estrogen receptor beta; *PR*, progesterone receptor; *AR*, androgen receptor. †Total patients except for inevaluable cases due to tissue loss or inadequate tissue in immunohistochemistry assay. ‡Chi-square test. §Japanese Classification of Gastric Carcinoma (3rd English edition) proposed by the Japanese Gastric Cancer Association (JGCA). ¶The 6th TNM Classification of Malignant Tumors proposed by the AJCC/UICC.

### ERα and AR expression correlates with favorable outcome in patients with gastric cancer

**Figure 3:**
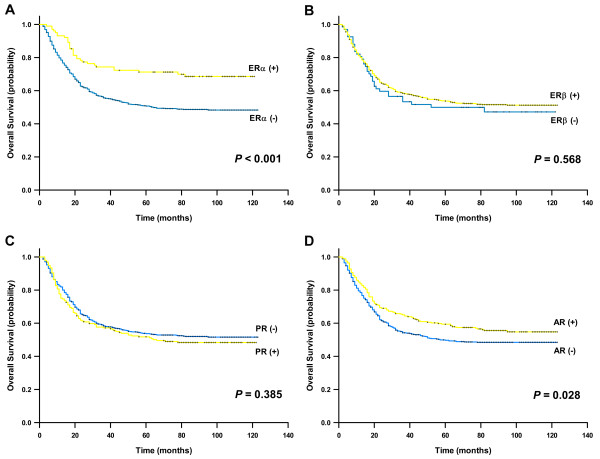
Kaplan-Meier survival curves according to (A) ERα, (B) ERβ, (C) PR, and (D) AR immunostaining (log-rank test).

Table 3 reports the findings from univariate and multivariate survival analysis in gastric cancer. Univariate analysis showed that the significant prognostic factors included age, tumor size, tumor grade, Lauren type, T classification, N classification, radical resection, TNM stage, ERα expression, and AR expression. For ERα-positive patients, the 5-year survival rate and median survival were 71.3% (95% CI, 62.5%-80.1%) and 81.5 months (range, 4–121 months), compared with 50.7% (95% CI, 47.0%-54.4%) and 43.0 months (range, 2–123 months) for ERα-negative patients, respectively. ERα-positive patients had a significantly better outcome than ERα-negative patients (*P* < 0.001; Figure 3A). The expression of ERβ and PR was not associated with the prognosis of gastric cancer patients (*P* = 0.568 for ERβ and *P* = 0.385 for PR; Figure 3B and 3C). For patients with AR-positive tumors, the 5-year survival rate was 59.4% (95%, 53.5%-65.3%) with a median survival of 71.0 months (range, 2–123 months), which took significant advantage over a 5-year survival rate of 49.7% (95% CI, 45.6%-53.8%) with a median survival of 40.0 months (range, 2–123 months) for patients with AR-negative tumors (*P* = 0.028; Figure 3D). However, only age, tumor size, T classification, N classification, and radical resection were retained in the multivariate Cox model, as shown in Table 3. The expression of ERα and AR were not independent prognostic factors for gastric cancer.

**Table 3:** Univariate and multivariate analysis of overall survival by Cox model in gastric cancer

**Variable**	**Univariate cox**			**Multivariate cox**		
	**HR**	**95% CI**	***P***	**HR**	**95% CI**	***P***
Sex: female *vs* male	0.896	0.728-1.103	0.302			
Age, years: ≤40 *vs* ≤50 *vs* ≤65 *vs* >65	1.262	1.127-1.412	<0.001	1.226	1.089-1.379	0.001
Tumor site: upper *vs* middle *vs* lower *vs* diffuse†	0.998	0.883-1.128	0.976			
Tumor size, cm: ≤2 *vs* ≤3 *vs* ≤5 *vs* >5	1.590	1.434-1.764	<0.001	1.149	1.023-1.291	0.019
Tumor grade: well *vs* moderate *vs* poor	1.540	1.287-1.841	<0.001	1.191	0.959-1.481	0.114
Lauren type: intestinal *vs* diffuse *vs* mixed	1.202	1.020-1.415	0.028	1.143	0.945-1.383	0.167
T classification: pT1 *vs* pT2 *vs* pT3 *vs* pT4‡	2.135	1.859-2.451	<0.001	1.490	1.263-1.757	<0.001
N classification: pN0 *vs* pN1 *vs* pN2 *vs* pN3‡	2.237	2.017-2.481	<0.001	1.733	1.543-1.947	<0.001
Radical resection: yes *vs* no	3.337	2.706-4.114	<0.001	2.053	1.641-2.570	<0.001
TNM stage: I *vs* II *vs* III *vs* IV‡§	2.306	2.064-2.576	<0.001			
ERα: positive *vs* negative	1.990	1.380-2.871	<0.001	1.159	0.797-1.685	0.441
ERβ: positive *vs* negative	1.107	0.779-1.573	0.572			
PR: positive *vs* negative	0.905	0.723-1.135	0.389			
AR: positive *vs* negative	1.265	1.023-1.564	0.030	1.072	0.858-1.340	0.541

*HR*, hazard ratio; *CI*, confidence interval; *ERα*, estrogen receptor alpha; *ERβ*, estrogen receptor beta; *PR*, progesterone receptor; *AR*, androgen receptor. †Japanese Classification of Gastric Carcinoma (3rd English edition) proposed by the Japanese Gastric Cancer Association (JGCA). ‡The 6th TNM Classification of Malignant Tumors proposed by the AJCC/UICC. §TNM stage was not included into multivariate Cox model to avoid repetition with T and N classifications.

## Discussion

In the present study, the expression profile and prognostic role of ERα, ERβ, PR, and AR in gastric cancer was determined in a large Chinese cohort. Our results showed that the four receptors were all expressed with decreased abundance in gastric tumors compared to adjacent normal tissues. All the four receptors were associated with high tumor grade and intestinal type, and the positive expression of ERα and AR also correlated with early TNM stage and thereby a favorable outcome.

Our findings are inconsistent with a few previous publications in which ERα, PR and AR were proposed as adverse factors whereas ERβ was deemed beneficial for gastric cancer patients [19,26-28]. Factually, substantial disagreement has been observed for several decades with regard to the expression and role of sex hormone receptors in gastric cancer. The conflicting findings may be partly attributed to heterogeneity in experimental methods, positivity criteria, sample size, and patient ethnicity. In particular, by various methods, the expression levels of sex hormone receptors even their presence, varies in a large range in gastric cancer. Furthermore, most of these investigations usually suffer from very small sample size and as a result, the conclusions on clinicopathological significance of sex hormone receptors in gastric cancer are often contradictory. Additionally, the failure to distinguish ERβ from ERα in earlier reports also contributes to conflicting results for ER.

To minimize the above-mentioned limitations, in this study, the expression of the four receptors was detected simultaneously at both mRNA and protein levels. More importantly, a set of tissue microarray containing tumors and corresponding normal tissues from 866 Chinese patients was employed. The largest sample size to date and the high uniformity of experimental conditions profiting from tissue microarray made our results more reliable and convincing.

Owing to the well establishment in breast cancer, ERs also are the best-studied sex hormone receptors in gastric cancer. Using a semi-quantitative RT-PCR, ERβ has shown a more preferential expression pattern than ERα in both gastric tumors and normal mucosa. Compared to the expression in normal gastric mucosa, ERα tends to increase in tumor tissues while ERβ declines [23,24]. With a real-time quantitative PCR, the presence of ERα and ERβ mRNA in all tumors and normal mucosa samples was demonstrated in our study. Moreover, the significant decrease of both ERα and ERβ mRNAs in tumors compared to in normal tissues was indicated, similar to the findings in another study from China [26].

Various expression patterns of ERα and ERβ proteins have been observed by several investigations. By IHC, a few studies have found no or sporadic staining of ERα in gastric tumors even in matched normal tissues, while strong ERβ staining in both cancer and their non-cancerous tissues [22,24,27,28]. Our results are not quite similar to these findings. ERα protein is indeed expressed in both normal mucosa (38.3%) and gastric tumors (12.0%) but with a very weak pattern, while ERβ protein is the predominant isoform of ER in both normal (97.3%) and cancerous (91.9%) tissues. Both ERα and ERβ proteins present a decreased expression pattern in gastric tumors compared to their corresponding normal tissues [26].

The presence of PR is detected in gastric cancer as early as ER, and the positive expression of PR ranges from none to all [9,10,12-21]. As far as AR is concerned, very little attention has been paid to AR in gastric cancer. Following the first detection of AR in 2 of 16 gastric cancer patients, a positive rate of 17.4% for AR nuclear staining in an immunohistochemical study of 86 cases was indicated [12,28]. In the present study, our results show a positive rate of 30.5% in normal mucosa and 23.3% in gastric tumors for PR, and 52.7% in normal tissues and 33.0% in tumors for AR.

When the localization of the four sex hormone receptors is concerned, a few earlier studies have revealed that they were solely expressed in the nuclei of gastric cancer cells [22,28]. However, a few recent studies also reported a cytoplasmic staining of ERα and a cytoplasmic/nuclear staining of ERβ in gastric cancer [26,27]. In the present study, unlike the typical nuclear expression in breast and prostate cancer tissues as positive controls (see Additional file 1: Figure S1), the four sex hormone receptors all presented a cytoplasmic/nuclear staining pattern (Figure 2). Particularly, ERα, PR and AR immunostaining was mainly localized in the cytoplasm while ERβ immunostaining was mostly detected in the nuclei.

Although our findings provided added information to the localization of sex hormone receptors in gastric cancer, agreement on the immunostaining location could not be finalized yet. Factually, the immunostaining location might be affected by the characteristics of antibody to some extent. In particular, different antibodies obtained from various clones usually result in inconsistent findings. For example, a sole nuclear immunostaining of ERα was observed with a mouse monoclonal antibody (clone ER88) [28] while a cytoplasmic expression pattern of ERα was detected with another mouse monoclonal antibody (clone 1D5) [26]. For ERβ, a sole nuclear expression was noticed with the use of a mouse monoclonal antibody (clone 14C8) [26] while with another mouse monoclonal antibody (clone PPG5/10), a cytoplasmic/nuclear expression was identified [27]. Therefore, it would be ideal to examine the immunostaining of the four sex hormone receptors synchronously by using a wide panel of antibodies obtained from various clones as many as possible. In the present study, only a single antibody for each receptor was used and the absence of other antibodies produced from various clones was the main limitation.

Nevertheless, based on the currently available evidence, the nuclear and cytoplasmic immunostaining patterns might indicate two different functional forms of sex hormone receptors in gastric cancer which were presumed to be dependent on the specific status of cancer cells. Seeing that the cytoplasmic expression of ERα and ERβ has also been observed in non-small cell lung cancer [32,33], the cytoplasmic expression of sex hormone receptors in these tumors derived from “nontarget” organs might suggest an independent or a novel mechanism involving in tumorigenesis distinct from the genomic signaling via nuclear forms typically occurred in breast cancer [33]. Certainly, more studies by use of novel antibody technology and special experimental techniques are warranted to carefully verify all of these assumptions in the future.

The associations between sex hormone receptors and clinicopathological factors of gastric tumors have been studied extensively. One of the most concerns is the expression difference of sex hormone receptors in both genders which might be a possible interpretation of male predominance among gastric cancer patients [34]. However, no significant difference is found between male and female patients in this study though slight higher positive rates in male patients are observed (Table 2), which suggested that the sex difference of gastric cancer incidence cannot attributed to sex hormone receptors at least.

In this study, the expression of four hormone receptors is all closely associated with tumor grade and Lauren type. Moreover, the positive immunostaining always indicates a high tumor differentiation and intestinal type (Table 2), and the positive rates of the four receptors in normal tissues are always significantly higher than that in corresponding tumors. Given sex hormone receptors are critical effectors of normal cell growth and differentiation, it is logical to assume that these receptors are also involved in the physiological maintenance of differentiation and function of gastric mucosa [35]. Together with the findings that sex hormone-receptor-negative tumors have a higher proliferative activity than sex hormone-receptor-positive tumors in human gastrointestinal tract adenocarcinomas [16], the abnormal expression or function of these receptors may be implicated in the pathogenesis of gastric cancer to some extent [5-7].

Besides tumor grade and Lauren type, the positivity of ERα and AR proteins also negatively correlates with advanced T and N classifications, and thereby correlates with an early TNM stage significantly (Table 2). Just as we expected, positive expression of ERα and AR correlates with favorable outcome for gastric cancer patients though they are not independent prognostic factors (Table 3). When ERβ and PR are concerned, no significant correlations are found between their expression and any clinicopathological characteristics except for tumor grade and Lauren type. Under this circumstance, the prognostic importance of ERβ and PR in gastric cancer is weak. In fact, so do our findings in the present study.

## Conclusion

Our results confirmed the presence of ERα, ERβ, PR, and AR in both gastric tumors and normal mucosa but their expression levels were extremely low except for the predominance of ERβ. The decreased expression pattern of the four receptors in gastric tumors and their associations with clinicopathological characteristics as well as overall survival indicate that the sex hormone receptors may be partly involved in gastric carcinogenesis. Together with the independent expression of the four receptors (Table 1) and the failure of hormone therapy for gastric cancer [36], the function and significance of sex hormone receptors in gastric cancer appears to be limited.

## Competing interests

The authors declare that they have no competing interests.

## Authors’ contributions

LG and JH performed experimental procedures, analyzed the data and drafted the manuscript. XZ, YJZ, GZY, YC, and JP performed experimental procedures, analyzed the data and reviewed the manuscript. JJW and XW designed the study, analyzed the data and drafted the manuscript. All authors read and approved the final manuscript.

## Pre-publication history

The pre-publication history for this paper can be accessed here:

http://www.biomedcentral.com/1471-2407/12/566/prepub

## Supplementary Material

Additional file 1 ** Figure S1.** Typical nuclear immunostaining of sex hormone receptors in breast and prostate cancer tissues as positive controls. Positive nuclear staining of (A) ERα, (B) ERβ, and (C) PR in breast cancer tissues, and positive nuclear staining of (D) AR in prostate cancer tissue is shown. Original magnification, × 400. (TIFF 1676 kb) Click here for file
