# Supplementary figures and images for: Expression profile and prognostic role of sex hormone receptors in gastric cancer
Source: BMC Cancer. 2012 Dec 2;12:566. doi: 10.1186/1471-2407-12-566 (PMC3517759; doi:10.1186/1471-2407-12-566)

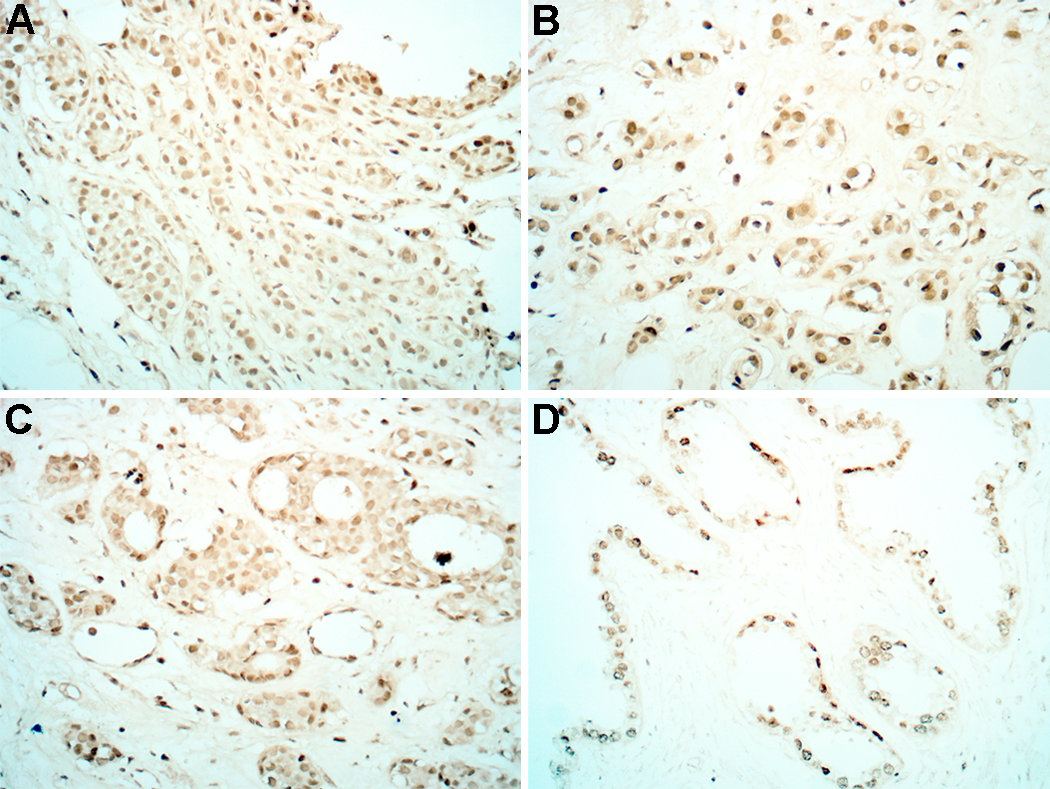

Supplement: Additional file 1 — Figure S1. Typical nuclear immunostaining of sex hormone receptors in breast and prostate cancer tissues as positive controls. Positive nuclear staining of (A) ERα, (B) ERβ, and (C) PR in breast cancer tissues, and positive nuclear staining of (D) AR in prostate cancer tissue is shown. Original magnification, × 400. (TIFF 1676 kb) [file 1471-2407-12-566-S1.tiff]
